# Effect of private insurance incentive policy reforms on trends in coronary revascularisation procedures in the private and public health sectors in Western Australia: a cohort study

**DOI:** 10.1186/1472-6963-13-280

**Published:** 2013-07-22

**Authors:** Shauna Trafalski, Tom Briffa, Joseph Hung, Rachael E Moorin, Frank Sanfilippo, David B Preen, Kristjana Einarsdóttir

**Affiliations:** 1 Centre for Health Services Research, School of Population Health, The University of Western Australia, 35 Stirling Highway, Crawley, Perth 6009, Western Australia; 2 Cardiovascular Research Group, School of Population Health, The University of Western Australia, 35 Stirling Highway, Crawley, Perth 6009, Western Australia; 3 School of Medicine and Pharmacology, Sir Charles Gairdner Hospital Unit and The University of Western Australia, 35 Stirling Highway, Crawley, Perth 6009, Western Australia; 4 Centre for Population Health Research, Curtin Health Innovation Research Institute, Curtin University, Kent Street, Bentley, Perth 6102, Western Australia; 5 Telethon Institute for Child Health Research, Centre for Child Health Research, The University of Western Australia, 100 Roberts Road, Subiaco 6008, Western Australia

**Keywords:** Health insurance, Coronary artery disease, Revascularisation procedures, Health policy

## Abstract

**Background:**

The Australian federal government introduced private health insurance incentive policy reforms in 2000 that increased the uptake of private health insurance in Australia. There is currently a lack of evidence on the effect of the policy reforms on access to cardiovascular interventions in public and private hospitals in Australia. The aim was to investigate whether the increased private health insurance uptake influenced trends in emergency and elective coronary artery revascularisation procedures (CARPs) for private and public patients.

**Methods:**

We included 34,423 incident CARPs from Western Australia during 1995-2008 in this study. Rates of emergency and elective CARPs were stratified for publicly and privately funded patients. The average annual percent change (AAPC) in trend was calculated before and after 2000 using joinpoint regression.

**Results:**

The rate of emergency CARPs, which were predominantly percutaneous coronary interventions (PCIs) with stenting, increased throughout the study period for both public and private patients (AAPC=12.9%, 95% CI=5.0,22.0 and 14.1%, 95% CI=9.8,18.6, respectively) with no significant difference in trends before and after policy implementation. The rate of elective PCIs with stenting from 2000 onwards remained relatively stable for public patients (AAPC=−6.0, 95% C= −16.9,6.4), but increased by 4.1% on average annually (95% CI=1.8,6.3) for private patients (p_difference_=0.04 between groups). This rate increase for private patients was only seen in people aged over 65 years and people residing in high socioeconomic areas.

**Conclusions:**

The private health insurance incentive policy reforms are a likely contributing factor in the shift in 2000 from public to privately-funded elective PCIs with stenting. These reforms as well as the increasing number of private hospitals may have been successful in increasing the availability of publicly-funded beds since 2000.

## Background

The Australian federal government introduced the private health insurance incentive policy reforms in 1997–2000 to encourage the uptake of private health insurance and increase the availability of beds in public hospitals [1,2]. From 1984, Australian residents had been able to access free treatment in public hospitals covered by national health insurance (public patients) and as a result, private health insurance membership among the Australian population began to decline [3]. These policy reforms were introduced as a response to this declining trend so that more patients would choose to be treated as private patients in either private or public hospitals at a subsidised cost through private health insurance [4,5].

The policy reforms were efficient in increasing private health insurance uptake, demonstrated by a rise in the percentage of population with private health insurance from 30% in 1999 to ~45% in 2001 [6]. Some evidence suggests that the reforms increased private health insurance utilisation as well, particularly for surgical in-patients episodes [7,8]. However, there is currently a lack of empirical evidence on the effect of the policy reforms on cardiovascular interventions and whether the reforms were successful in relieving pressure on access to coronary interventions in public hospitals. Such information is particularly important as cardiovascular diseases are the most expensive group of diseases in Australia [9]. Coronary heart disease (CHD) alone accounted for 31% of the total cardiovascular disease cost in 2004–5 or $1.8 billion [9], with almost three-quarters of the cost due to CHD spent on hospital-admitted patients [9]. This high cost of cardiovascular interventions in hospitals can mainly be attributed to coronary artery revascularisation procedures (CARPs) as they are the primary procedural intervention for stable and unstable CHD in Australia. These procedures include coronary artery bypass grafting (CABG) and percutaneous coronary intervention (PCI), with or without stenting [10].

The high investment in private health insurance by the Australian federal government and the high economic burden of CARPs on the health care system, emphasises the need to investigate this important issue. The objective of this study was therefore to assess whether the increased uptake of private health insurance in Australia after the implementation of the Federal government’s policy initiatives (around the year 2000) resulted in more CARPs being performed in the private sector and if so, what impact it had on CARPs in the public sector and whether trend were consistent across age and socioeconomic status (SES).

## Methods

### Study design

In this study, rates of emergency and elective CARPs were calculated from 1 January 1995 to 31 December 2008 in Western Australia (WA). The rates were stratified for publicly and privately funded patients.

The procedure rates were calculated from the yearly procedure counts in our data (numerators) and annual population figures for 35+ year old individuals resident in WA (denominators) based on 5-yearly census data published by the Australian Bureau of Statistics [11]. When we analysed elective PCIs with stenting by age and SES group, both numerators and denominators were stratified according to the respective groups.

### Study sample

All CARPs in WA were identified using routinely-collected administrative health data from the WA Hospital Morbidity Data Collection at the Department of Health. The data fields included up to 11 procedures, 21 diagnoses, dates and types of admissions (emergency or elective) and separations for all inpatient episodes in all WA public and private hospitals.

The study cohort included all individuals aged 35 years and over who had their index (or first) CARP at a WA hospital between 1 January 1995 and 31 December 2008. Hospital admissions dating back to 1 January 1985 were available to determine prior CARPs using the International Classification of Disease (ICD) codes for the procedures, versions 9 and 10, as shown in Table 1. First-ever CARP was defined on the basis of no coronary revascularization procedure recorded on a WA hospital admission in the previous 10 years. CARPs performed on an elective admission (elective CARPs) were distinguished from emergency CARPs, defined as a CARP performed during a non-elective (emergency) cardiovascular admission.

**Table 1:** CARPs ICD9 and 10-AM procedure codes

	**ICD-9 and ICD-9-CM**	**ICD-10-AM**
CABG	36.10 – 36.19	38497, 38500, 38503, 90201
PCI without stenting	36.01, 36.02, 36.05	35304-00, 35305–00, 38303–00, 38300-00
PCI with stenting	36.06, 36.07	35310 (−00, -01, -02), 3830600–00, 38306–01, 38306-02

The hospital data also contained information on the Index of Relative Socio-Economic Disadvantage (IRSD) [12] and the funding source (private or public) of the patient at the time of their CARP. The IRSD values are based on information on household income, educational attainment and occupation from the Australian Census conducted every five years and assigned to each collection district area, about 250 households, in the state. The IRSD values were divided into two groups with those living in the lowest 40% SES areas classified as low SES with others classified as living in a high SES area. Patient funding source was binary; those who elected to be treated as public patients and those who elected to be treated as private patients, independent of whether the hospital where they underwent treatment was classed as a public or private institution. Public patients consisted of those who were insured under the Australian Health Care Agreement and Reciprocal Health Care Agreements. Private patients included those who were funded through private health insurance or were self-funded.

### Statistical analysis

The average annual percent change (AAPC) and 95% confidence intervals for the trend in procedure rate during 1995–2008 was calculated using the Joinpoint Regression Program Version 3.4.3 (http://surveillance.cancer.gov/joinpoint/). The joinpoint regression analysis was used to identify points at which statistically significant changes in temporal trend occurred and to calculate the annual percent change in each segment. The average percent change in procedure rate per year across the joinpoint segments was then calculated for the entire study period (1995–2008) as well as two separate time periods; 1995–2000 and 2000–2008. A negative AAPC indicated a decreasing trend on average whereas a positive AAPC indicated an increasing trend on average. All other analyses were performed using the statistical software SAS version 9.3 (SAS Institute Inc., Cary, NC, USA).

## Results

From January 1995 to December 2008 there were 34,423 index CARPs performed in WA hospitals, including 14,089 (41%) classified as emergency CARPs and 20,334 (59%) as elective. From the index CARP cases, 57.4% were publicly funded patients and 42.6% were privately funded, with 94% of the public patients treated in public hospitals and 72% of the private patients treated in private hospitals. Privately funded patients were 40% pre-2000, which increased to 44% post-2000.

The proportion of patients treated in public hospitals - whether privately or publicly funded – was lower for pre-2000 than post-2000, whereas the proportion of private hospital patients increased regardless of their funding status (Table 2). This trend was particularly evident for patients undergoing elective CARPs as the proportion of public patients treated in public hospitals declined from 51.1% in the period prior to year 2000 to 39.4% after year 2000 with the proportion of private patients treated in public hospitals showing a similar pattern in terms of absolute percent decrease (15.1% to 5.7%).

**Table 2:** Proportion of index CARP patients (n=34,423), elective (n=20,334) and emergency (n=14,089) by funding source, hospital type and time period

	**Before 2000**	**After 2000**	**Total**
All	N (%)	N (%)	N (%)
Public patient in public hospital	6,363 (58.9)	12,275 (52.0)	18,638 (54.1)
Public patient in private hospital	115 (1.1)	1,000 (4.2)	1,115 (3.2)
Private patient in public hospital	1,696 (15.7)	2,422 (10.3)	4,118 (12.0)
Private patient in private hospital	2,621 (24.3)	7,931 (33.6)	10,552 (30.7)
Elective			
Public patient in public hospital	3,787 (51.1)	5,092 (39.4)	8,879 (43.7)
Public patient in private hospital	59 (0.8)	550 (4.3)	609 (3.0)
Private patient in public hospital	1,121 (15.1)	742 (5.7)	1,863 (9.2)
Private patient in private hospital	2,441 (33.0)	6,541 (50.6)	8,983 (44.2)
Emergency			
Public patient in public hospital	2,576 (76.1)	7183 (67.1)	9,759 (69.3)
Public patient in private hospital	56 (1.7)	450 (4.2)	506 (3.4)
Private patient in public hospital	575 (17.0)	1680 (15.7)	2,255 (16.0)
Private patient in private hospital	180 (5.3)	1389 (13.0)	1,569 (11.1)

CARP=coronary artery revascularisation procedure.

**Figure 1:**
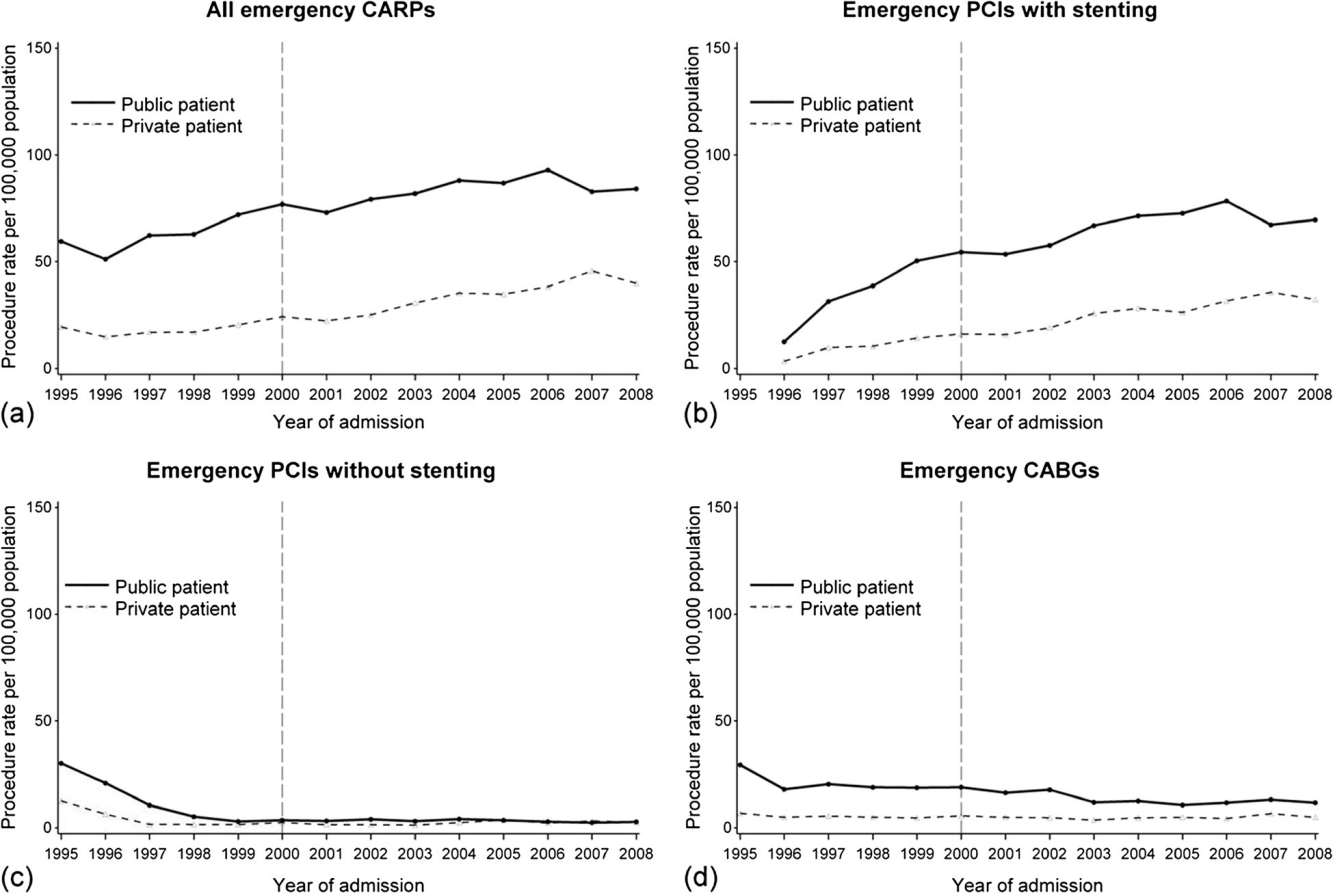
**Annual rates of emergency coronary artery revascularisation procedures (CARPs) for public (n=10265) and private (n=3824) patients in WA during 1995–2008 for (a) all CARPs (b) percutaneous coronary intervention (PCI) with stenting (c) PCI without stenting and (d) coronary artery bypass grafting.** The vertical line represents the end of the introduction period of the private health insurance incentive reforms.

The rate of all emergency CARPs increased steadily before and after the year 2000 (Figure 1a) for both public (60% in 1995 to 84% in 2008) and private patients (19% to 40%). The rate of PCIs with stenting showed a similar escalating trend pattern to all CARPs (Figure 1b). The rate of emergency PCIs without stenting (Figure 1c) decreased before year 2000 similarly in both groups (p_difference_=0.6) and then remained relatively stable at a low rate from 2000 onwards. The rate of all emergency CABGs declined in public patients before and after 2000 (AAPC= −5.8, 95% CI=−7.2,-4.4) and remained relatively steady at a low rate throughout in private patients (AAPC=0.2, 95% CI=−2.8,3.3).

**Figure 2:**
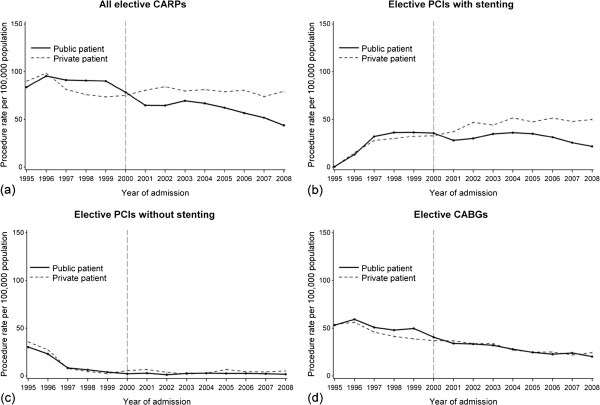
**Annual rates of elective coronary artery revascularisation procedures (CARPs) for public (n=9488) and private (n=10846) patients in WA during 1995–2008 for (a) all CARPs (b) percutaneous coronary intervention (PCI) with stenting (c) PCI without stenting and (d) coronary artery bypass grafting.** The vertical line represents the end of the introduction period of the private health insurance incentive reforms.

The rate of all elective CARPs decreased for both public and private patients during 1995–2008 from 84% to 44% and 90% to 79% respectively (Figure 2a). This decrease was similar between the two groups before 2000 (p_difference_=0.9), but from 2000 onwards the decrease attenuated for private patients (p<0.0001). The rate of elective PCIs with stenting increased for both public and private patients during 1995–2000 (p_difference_=0.9), but from 2000 onwards the rate increased only for private patients by 4.1% (95% CI=1.8,6.3) on average annually, while it remained stable for public patients (AAPC= −6.0, 95% C= −16.9,6.4) (p_difference_=0.04). Elective PCIs without stenting decreased in both public and private patients before the year 2000, and thereafter remained steady at a low rate in both groups (p_difference_=0.6). Elective CABGs decreased throughout the study period with no apparent differences between public and private patients.

**Figure 3:**
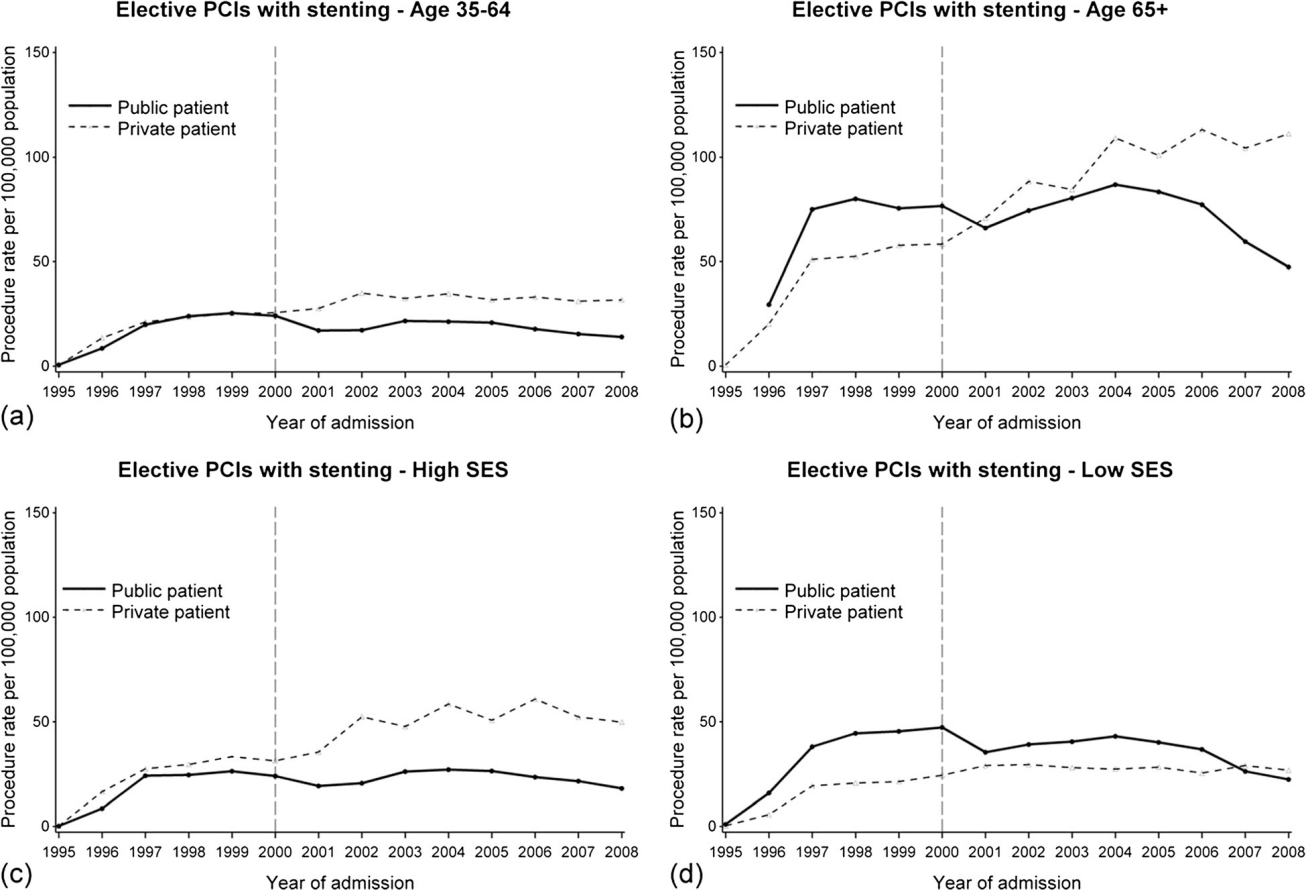
**Annual rates of elective percutaneous coronary intervention (PCI) with stenting stratified by funding source in WA during 1995–2008 for (a) people aged 35–64 years (b) people aged 65 years and over (c) people residing in high SES areas (d) people residing in low SES areas.** The vertical line represents the end of the introduction period of the private health insurance incentive reforms.

Figure 3 shows elective PCIs with stenting in public and private patients stratified by age 35–64 and 65+ years and by high and low SES. Post year 2000, the rate of elective PCIs with stenting in public patients remained relatively unchanged irrespective of age group and SES. For private patients post-2000, the rate of elective PCIs with stenting increased more steeply in people aged 65+ years (AAPC=7.2%, 95% CI=2.9,11.5) than their younger counterparts (AAPC=1.7, 95% CI=−0.2,3.7) as well as for private patients living in high SES areas (AAPC=5.1%, 95% CI=−0.6,11.0), compared with low SES areas (AAPC=0.8%, 95% CI=−0.4,2.0).

## Discussion

Our results describe the trend in the rate of emergency and elective CARPs in WA for public and private funding sources before and after the introduction of the Federal government’s private health insurance incentive policy reforms in 2000. The rates of emergency procedures – which included mainly PCIs with stenting - increased throughout the study period for both public and private patients. Elective PCIs with stenting, however, increased only for privately funded patients following the introduction of the private health insurance incentive policy reforms. This rate increase was only evident for people aged 65+ years and people living in high SES areas.

Despite the steady increase in emergency PCIs with stenting for both public and private patients throughout the study period, our results showed that the majority of these procedures continue to be performed in the public sector based on the absolute procedural rates. Over 80% of patients requiring emergency CARPs are still treated in public hospitals as a public or private patient. As such, there appears to be a core group of patients that will continue to require emergency CARPs, predominantly PCIs with stenting for management of acute coronary syndromes, and who are not influenced by private health insurance policy reforms to purchase private health. However, there was a minor impact in the sense that the proportion of emergency CARPs which were performed in private hospitals increased from 7% to 17.2% before and after year 2000. This increase in rate of emergency CARPs is likely to be attributed to many factors, including an ageing population, evidence-based practice of primary PCI for ST segment elevation myocardial infarction [13], and current clinical guidelines that recommend an early invasive management strategy over an initially conservative strategy for acute coronary syndrome cases [14,15]. Our results also showed a declining trend for elective CABGs in both public and private patients. This predated the policy reforms and appears not to have been influenced by their introduction.

In our study, the rate of elective PCIs with stenting increased only for private patients from 2000 onwards. Notwithstanding controlling for increased availability of PCI since the introduction of the private health insurance incentive policy reforms, this initiative appears to have been successful in shifting elective PCIs with stenting from the public to the private sector particularly in the older age group which traditionally has been the group who are most disadvantaged in terms of access to elective surgery in public hospitals. Interestingly, the findings from this study also indicate that the increased rate of PCIs with stenting for private patients from 2000 onwards was only evident for people living in high SES areas. Therefore, it seems possible that mainly Western Australians with sufficient discretionary income were able to respond to the increased affordability of private health insurance membership following the introduction of the private health insurance policy reforms by shifting from the public to the private sector. The reason why there was not a comparable increase in rate of elective PCIs with stenting for private patients living in low SES areas is unclear because the rates of cardiovascular disease would be expected to be higher in people living in low SES areas and suggests that a person’s SES and the area in which he/she lives has a significant effect on access to health care regardless of access to private health insurance.

The shift from publicly funded to privately funded elective PCIs with stenting from 2000 would likely have relieved the economic pressure on the public sector. However, due to the high cost of these procedures in the private sector and the increasing investment of the federal government towards the private sector, the shift may in fact not have improved the economic burden on the public purse. For example, the private health insurance incentives that were introduced in 2000 involved a 1% tax-penalty for high income earners without health insurance, a 30% tax rebate on insurance premiums and a 2% premium penalty pa for those who entered after the age of 30 [1,16]. The 50% increase in private health insurance membership following the introduction of these reforms has been attributed primarily to the introduction of the premium penalty as the 30% premium rebate was reported to increase private health insurance coverage by only 1% from 1998 to 1999 [17,18]. It therefore seems that the most costly policy reform, i.e. the 30% rebate, did not do the trick. However, because of the 30% rebate, government funding for patients undergoing invasive cardiac procedures (F42B) was lower for public patients ($1,424 per episode) than for private patients ($2,254) in 2005–6 [19]. In addition, since the premium rebate is 35% for people aged 65–69 years and 40% for people aged 70 years and over, the government funded a greater proportion of private patient costs for the older age groups [19]. Furthermore, the costs of PCI procedures have been found to be more than twice as high in the private sector compared with the public sector [20]. As a result, although it has been argued that the policy reforms were successful in relieving economic pressure on the public sector, our results present some evidence to suggest that this may not necessarily have been the case.

What appears to be more likely than a reduction in the economic pressure on public hospitals is that the policy reforms may have increased the availability of beds in public hospitals. The vast majority of public patients included in this study were treated in public hospitals and the proportion of patients undergoing CARPs in public hospitals decreased significantly from pre-2000 to post-2000. This decreasing trend was particularly evident for elective procedures. This supports our results suggesting that when private health insurance became more affordable in the year 2000, it was the elective cases that tended to transfer to the private sector. What also seems to have made this shift easier is that the number of private hospitals in Australia had been increasing since the 1990. For example, there was a 16% increase in the number of private hospitals in Australia during 1990–2000, whereas public hospital numbers increased by only 3% over the same time [21].

The strength of this study is reflected in the use of 14 years of routinely-collected hospital inpatient information for the entire WA population. It is a statutory requirement that the Department of Health records information on all hospital admissions and separations from all public and private hospitals in the State. The hospital data collection has undergone stringent quality assessments by the Department of Health and validation studies have confirmed the accuracy of the coding [22,23]. Despite these strengths, some limitations require acknowledgement. Firstly, we were not able to quantify or account for the increase in catheter laboratories in metropolitan Perth or the increase in cardiologists who can perform PCIs with stenting. We can thus not say for certain whether the shift from publicly-funded elective PCIs with stenting to privately-funded elective PCIs with stenting around 2000 was due to the private health insurance incentive policy reforms alone. Secondly, we cannot distinguish between bare stents and drug eluting stents. This is a constraint since drug eluting stents were more commonly used in the private sector. They were also more expensive and delivered better near-term or early procedural outcomes compared with bare stents. And thirdly, our data showed that not all private patients were treated in private hospitals, a small proportion of the privately funded index CARP patients were treated in a public hospital.

## Conclusions

This study investigated whether the increased uptake of private health insurance following the Australian private health insurance incentive policy reforms introduced in 2000 resulted in an increased rate of CARPs in patients electing for private care. Our results show a rise of privately funded elective PCIs with stenting after 2000, but a decline in publicly funded elective procedures. As such, these reforms may have been successful in improving access to elective revascularization procedures overall and reducing pressure on public hospitals for the interventional management of acute coronary syndromes in Western Australia. However, due to the high cost of these procedures in the private sector and the increasing investment of the federal government towards the private sector, the shift may not have improved the economic burden on the public purse.

## Abbreviations

AAPC: Average annual percent change; PHI: Private health insurance; CARP: Coronary artery revascularisation procedure; WA: Western Australia; HMDC: Hospital morbidity data collection; CABG: Coronary artery bypass grafting; PCI: Percutaneous coronary intervention; CHD: Coronary heart disease; IRSD: Index of relative socio-economic disadvantage.

## Competing interests

The authors declare that they have no competing interests.

## Authors’ contributions

ST analysed the data and wrote the article. TB and JH supervised the work and gave clinical advice on cardiovascular disease. RM, FS and DBP gave statistical input and advice on Australian health care policy and analysis of administrative data. KE initiated the research, designed the study, and interpreted the data. All authors revised the paper critically for important intellectual content and approved it to be published.

## Pre-publication history

The pre-publication history for this paper can be accessed here:

http://www.biomedcentral.com/1472-6963/13/280/prepub
